# Editorial: Recent insights into the role of hormones during development and their functional regulation

**DOI:** 10.3389/fendo.2024.1454714

**Published:** 2024-07-19

**Authors:** Shaojun Liu

**Affiliations:** State Key Laboratory of Developmental Biology of Freshwater Fish, College of Life Sciences, Hunan Normal University, Changsha, Hunan, China

**Keywords:** endocrinology, hormones, development process, regulation, gene

Hormones play a critical role in the complex processes of development in organisms. From embryogenesis to adulthood, hormones regulate multiple biological processes such as growth, metabolism, and reproduction (1). Hormonal regulation originates from tissues that secrete hormones directly into the circulatory system. Once binding to their receptors, hormones can activate the signaling pathways in target cells and modulate gene expression (2). The timing and level of hormone release are meticulously controlled by various feedback mechanisms, ensuring that homeostasis is maintained across varying internal and external conditions (3). Understanding the function of hormones involves not only their synthesis and secretion but also the exploration of the signaling pathways they activate and the genetic transcription mechanisms they control.

This Research Topic is entitled “Recent Insights into the Role of Hormones during Development and Their Functional Regulation”. It complied studies on structure, expression and effects of endocrinology-related gene, regulating growth and development process. This topic includes five contributions in total, four original articles and one mini-review, covering various aspects of endocrine regulation during development.

Molting in crustaceans is a biological process regulated by ecdysone hormones. To delve into the genetic interactions between ecdysone and its associated genes in molting regulation, Chen et al. investigated the expression patterns of the *EcR* and *RxR* genes in the Chinese mitten crab (*Eriocheir sinensis*) during the molting cycle. They also used co-immunoprecipitation and luciferase assays to explore the genetic interactions of these genes in the molting process. The study revealed that *RxR* was more stably expressed and at higher levels than *EcR* throughout the molting cycle. In contrast, the expression of *EcR* increased sharply during the pre-molt stage. Co-immunoprecipitation confirmed the interaction between EcR and RXR, while the dual-luciferase assay demonstrated that their heterodimer complex strongly activated the transcription of the ecdysone pathway’s key gene *E75*. These results offer new insights into the endocrine control of molting and molecular mechanisms of the ecdysone signaling pathway in crustaceans.

Ghrelin is well-known as a digestive endocrine hormone that regulates individual growth and feeding. Zhong et al. investigated the expression patterns of the *ghrelin* gene in the stomachs of hybrid tilapia and their parents using real-time quantitative PCR and pyrosequencing techniques. Overall, the expression of *ghrelin* in the stomachs of hybrid tilapia was significantly higher than that in the parents. At the allelic level, the *ghrelin* gene in hybrid tilapia exhibited a maternal dominant expression pattern in the stomach, which was not affected by the states of hunger or fullness. By evaluating *cis* and *trans* regulatory effects through overall gene and allelic expression results, it was found that *ghrelin* in hybrid tilapia was mainly influenced by compensating *cis* and *trans* effects. These results provide a basis for exploring the mechanisms underlying heterosis in tilapia.

In the mini-review, Aref et al. reviewed the new knowledge about the function of hormones in development. They reviewed the changes of hormones during different periods (including fetal development, childhood growth, puberty and after puberty) and their regulatory effects on physiological characteristics such as growth and sex differentiation. This review reinforces the new reports suggesting that classic hormones such as sex hormones have been systematically investigated during ontogenesis. However, the mechanisms and the network of hormones are still far away from revealing.

Energy metabolism homeostasis is closely related to endocrine regulation. The melanocortin-3 and -4 receptors (MC3R and MC4R) are directly involved in this regulation. In an original article, Huang et al. investigated the gene expression characteristics of *mc3r* and *mc4r* and their function in red crucian carp by knockout study using the CRISPR/Cas9 system. The results showed that the two genes were highly expressed in brain. The *mc4r^+/-^* fish had better growth performance and more visceral fat mass while the *mc3r* ^+/-^ fish showed no significance differences compared to wild type fish. In addition, the *mc4r^+/-^* fish had more visceral fat mass than that in the *mc3r* ^+/-^ and wild type fishes. RNA-seq showed the pathways that related to lipid accumulation and growth were changed in *mc3r* ^+/-^ and *mc4r^+/-^* fishes. The study elucidated the growth and lipid regulation by MC4R in red crucian carp, offering potential target for endocrine regulation of productive traits in fishes.

Fish distant hybridization can generate hybrids with advanced traits, which are extensively cultivated in aquaculture. Qing et al. analyzed the gene expression in Growth Hormone (GH)/Insulin-like growth factor 1 (IGF-1) axis from the hybrid triploid crucian carp and its parents. The results indicated that most of the genes including *GHR*, *IGF1*, *IGF2*, and *IGF-1Ra* showed higher expression in the triploid crucian carp than that in red crucian carp and common carp. In addition, the gene expression pattern analysis suggested that 5 genes were transgressive upregulation and 11 genes showed nonadditive expression in triploid crucian carp. These evidences provided new insight into the genetic and endocrinological basis for heterosis in fish generated by distant hybridization.

In summary, this Research Topic for Frontiers in Endocrinology: Recent Insights into the Role of Hormones during Development and Their Functional Regulation presented an overview of the current advances in hormone regulation in animals. The compiled works highlight the importance of hormones in the developmental process and their regulatory mechanisms.

## Author contributions

SL: Writing – original draft, Writing – review & editing.
